# Training medical students’ diagnostic reasoning skills using multivariate analysis

**DOI:** 10.1186/s12909-026-08604-1

**Published:** 2026-01-30

**Authors:** Fábio A. Schaberle, João Pestana, Luiz M. Santiago, Alberto A. C. C. Pais

**Affiliations:** 1 Department of Chemistry, CQC-IMS, University of Coimbra, Coimbra, 3004-535 Portugal; 2 Faculty of Medicine, University of Coimbra, Coimbra, 3000-548 Portugal; 3 Centre for health studies and investigation of the University of Coimbra (CEISUC), University of Coimbra, Coimbra, 3004-512 Portugal

**Keywords:** Medical reasoning, Differential diagnosis, Self-directed learning, Multivariate analysis, Digital competencies

## Abstract

**Background:**

Contemporary medical education must address both information overload and the need to develop robust diagnostic reasoning skills for common and less prevalent diseases. While traditional methods remain foundational, integrating self-directed learning skills is critical to prepare future clinicians. To face these challenges, we propose employing multivariate analysis as a structured approach to diagnostic training, enabling students to systematically evaluate symptoms and signs, environmental contexts, and risk factors. This method is adaptable to both classroom and self-directed learning, offering a pragmatic tool for training clinical decision-making, reinforcing self-directed learning by integrating data science with diagnostic reasoning training.

**Methods:**

The proposed method involves creating a structured database of diseases, their core symptoms and signs, and associated risk or environmental factors. Students then use this database as input for multivariate analysis (principal component analysis, PCA, and hierarchical cluster analysis, HCA) through guided R scripting exercises. By entering relevant symptoms and risk factors, learners can simulate diagnostic processes. The analysis generates: (1) biplots visualizing relationships between clinical features and diseases, and (2) dendrograms clustering clinically related conditions for comparative analysis.

**Results:**

We implemented an example of database using ICPC-2 nomenclature, containing diseases with associated symptoms and signs and risk factors. Diagnostic simulations demonstrated the application of the method across diverse clinical presentations, generating correlation plots and dendrograms for each case analysis. The outputs effectively showed both common diagnoses and rare conditions suggested by input symptoms and signs, prompting for the student to consider additional diagnostic factors. Notably, the system highlighted cases where uncommon diagnoses warranted further patient history (e.g., travel to endemic regions, family history), demonstrating its potential to train diagnostic reasoning by expanding students’ differential diagnosis considerations.

**Conclusions:**

The process of building disease databases and performing multivariate analysis appears valuable for medical education, offering students opportunities to explore disease-symptom relationships while developing complementary programming skills. This approach may help medical students recognize how similar symptoms can lead to diagnostic challenges, encouraging more comprehensive patient evaluation. The methodology presented here could serve as a potential teaching tool for medical curricula, combining diagnostic reasoning practice with data analysis skills in a clinically relevant framework.

**Supplementary Information:**

The online version contains supplementary material available at 10.1186/s12909-026-08604-1.

## Background

The rapid expansion of medical knowledge presents both opportunities and challenges for undergraduate medical education, particularly in developing diagnostic reasoning skills. Although access to medical information has expanded dramatically, educators must develop strategies to help students - particularly those training for primary care roles - effectively translate this knowledge into clinical practice, given that family physicians often serve as patients’ first point of contact [1]. Current approaches increasingly incorporate computational tools, such as virtual patients [2] and probabilistic simulators [3], to bridge this gap. These methods align with established diagnostic frameworks that emphasize information identification, hypothesis testing, and clinical interpretation [4].

Digital learning technologies, including AI-driven platforms and curated internet resources, show particular promise for both classroom and self-directed learning environments [5]. However, their educational value depends fundamentally on thoughtful instructional design - without proper scaffolding, students may become overwhelmed by information overload or fail to develop robust clinical decision-making skills. This reality highlights the need for structured, technology-enhanced learning approaches that maintain strong pedagogical foundations while promoting clinical reasoning development. To address this need, we propose an innovative exercise integrating database construction with multivariate statistical analysis. In this approach, students: (1) compile a focused clinical database encompassing diseases, their core symptoms, and associated risk factors; and (2) implement Principal Component Analysis (PCA) and Hierarchical Cluster Analysis (HCA) through guided programming exercises.

The proposed method addresses three core educational objectives: (1) improving familiarity with both common and rare disease presentations through active database curation, (2) cultivating essential data analysis and programming competencies, and (3) strengthening diagnostic reasoning skills through multivariate visualization tools. This novel approach to enhancing medical students’ diagnostic reasoning skills through the use of multivariate analysis (PCA and HCA) applied to symptom–disease databases. Its key strengths include the originality of integrating statistical methods into diagnostic training, the dual emphasis on both clinical reasoning and digital/data literacy, and the provision of practical examples with reproducible R code. The concept aligns well with contemporary educational priorities in medicine, especially the need to foster self-directed learning and computational competencies. Specifically, the biplots and cluster dendrograms provide immediate visual feedback, helping students recognize clinically significant patterns among symptoms, diseases, and risk factors. Designed for flexibility, this framework supports both instructor-led sessions and independent learning while maintaining rigorous clinical relevance.

Principal Component Analysis (PCA) is an unsupervised multivariate method that identifies underlying correlations between variables - in this context, diseases and their clinical features. As an unsupervised approach, it requires no a priori assumptions, deriving patterns solely through statistical relationships [6]. PCA promotes dimensionality reduction, acting by a transformation of variables that intends to preserve variability. It provides loadings, corresponding to the coefficients that translate the original variables in the transformed ones. It also provides scores, the representation of the original objects in the new coordinate system and eigenvalues which are a measure of the variability corresponding to each component, i.e., a measure of relevance. PCA results are often represented as biplots, a two-dimensional representation where both scores and loadings are visible. This allows to inspect positioning (scores), and the role of variables.

Hierarchical Cluster Analysis (HCA) complements this by grouping similar entities into dendrogram structures, where inter-cluster distances quantitatively represent phenotypic similarity [7]. This method, in its associative form, evolves from a situation in which all objects are separated to one in which they are gathered in a single cluster, justifying the term “hierarchical”. In favourable situations, clusters may be identified upon inspection of the dendrogram. As such, the information from dendrograms and biplots is compatible.

Together, these two methods enable systematic exploration of clinical relationships: PCA reveals how symptoms and risk factors covary across diagnoses, while HCA clusters diseases by shared clinical symptoms. For instance, when analysing diseases as observations characterized by symptoms and risk factors as variables, PCA quantifies their associations, while HCA visually organizes diseases into clinically meaningful groupings based on symptoms similarity. Note that, rather than assessing the outcomes of the exercise, we present it, for the time being, as a practical resource for use in the classroom. We highlight its potential value as an educational tool that can serve as a foundation for future evaluation studies.

## Methods

### Database

Students can construct the database using a spreadsheet framework, with diseases as individuals (rows) and associated symptoms/risk factors as variables (columns). As exemplified in Table 1, disease nomenclature adheres to ICD-10 codes, while symptom classification follows the standardized ICPC-2 (WHO-recognized) keywords. Risk factors are similarly encoded using concise descriptors. Though the database example used in this work emphasizes three hallmark symptoms and two key risk factors per disease (Table 1), the framework intentionally maintains flexibility for curricular adaptation or student choice. The complete reference database, having 133 diseases, used in this manuscript is available in Supplementary Materials. Note that using the standardized ICPC-2 system (WHO-recognized), provides validated keywords and definitions. This approach is intended not only to reduce the likelihood of incorrect disease–symptom associations but also to promote the consistent use of authoritative medical sources.

**Table 1:** Representative database structure with symptom and risk factor keywords

Disease	Symptom1	Symptom2	Symptom3	Risky factor1	Risky factor2
Type 1 diabetes mellitus	Thirsty	Weight Loss	Polyuria	Family History	Ethnicity
Type 2 diabetes mellitus	Thirsty	Weight Loss	Polyuria	Obesity	Age
Obesity	Body Fat	Type 2 Diabetes Mellitus	Cardiovascular Disease	Family History	Diet

### Computational script

The PCA and HCA analyses can be implemented using R language within the RStudio integrated development environment [8]. R provides built-in functions for both multivariate techniques The following packages were used in the examples: “FactoMineR” [9] (to perform PCA and HCA), “factoextra” [10] (to visualize the results), “ggplot2” [11], and “devtools” [12]. For beginners, comprehensive tutorials on implementing these analyses in R are available [13]. The core workflow involves: (1) data import using read.csv(), (2) data pre-processing with prcomp() (PCA) or hclust() (HCA), and (3) visualization via fviz_pca() (from factoextra) or plot() for dendrograms. The complete annotated code used in this study is provided in Supplementary Materials. Students may optionally leverage AI-assisted coding tools for initial script generation.

The computational workflow follows three key stages: First, the script imports the disease database and prompts for user inputs (symptoms/risk factors). Next, it constructs a binary matrix where matched clinical features are encoded as 1 (present), or frequency-weighted number, and non-matches as 0. For frequency-weighted analysis, a routine implementation replaces keywords with their occurrence counts across the database (e.g., ‘fever’ might score 15 if appearing in 15 diseases). This numeric transformation enables quantitative multivariate analysis. Finally, the script executes PCA and HCA using the processed matrix, generating correlation plots and dendrograms as diagnostic decision aids. The main algorithm steps workflow is given below.

**Figure:**
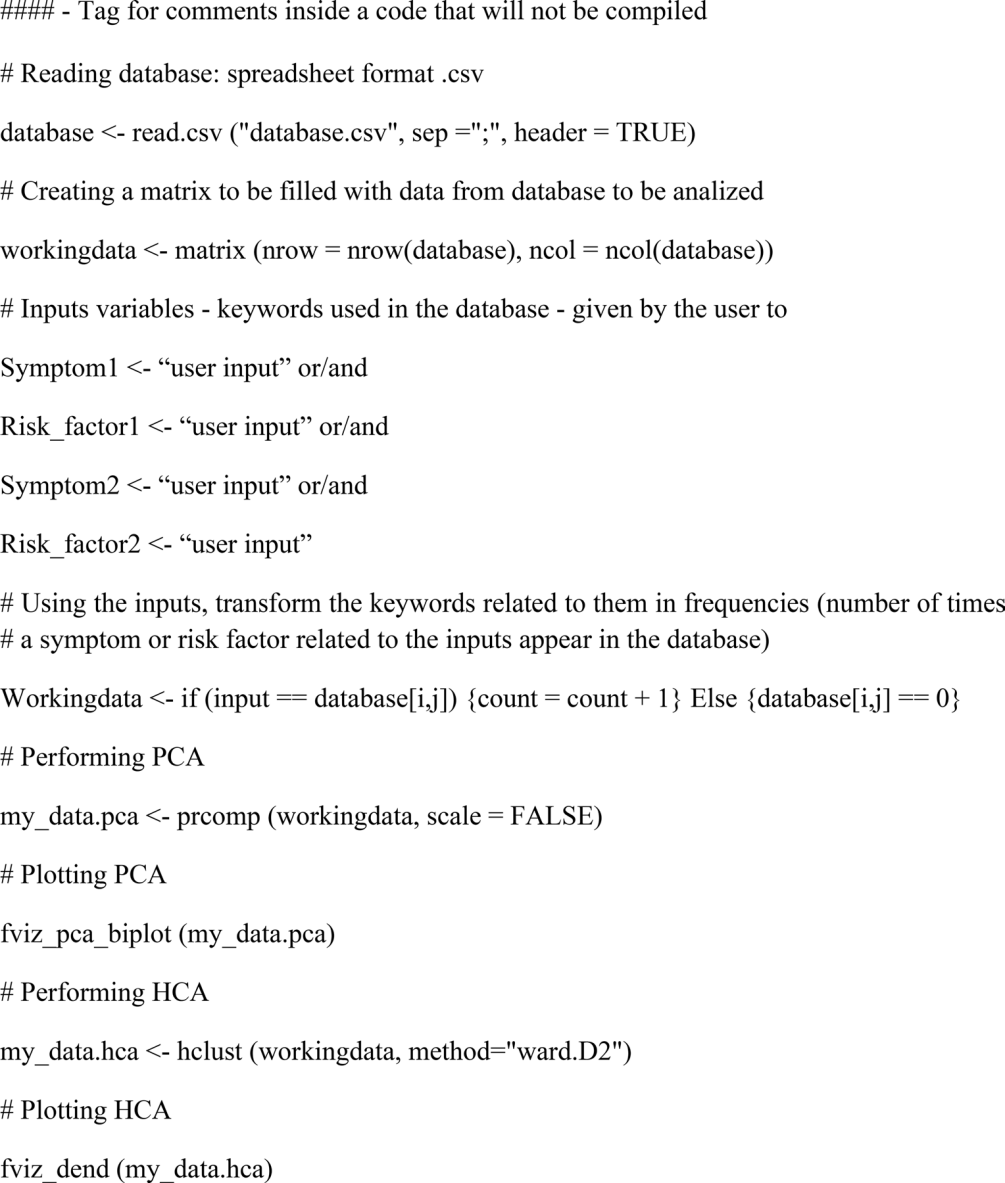
**Algorithm 1.** Main algorithm workflow

## Results

### Example 1- inputs: symptom1: myalgia; symptom2: joint pain

**Fig. 1:**
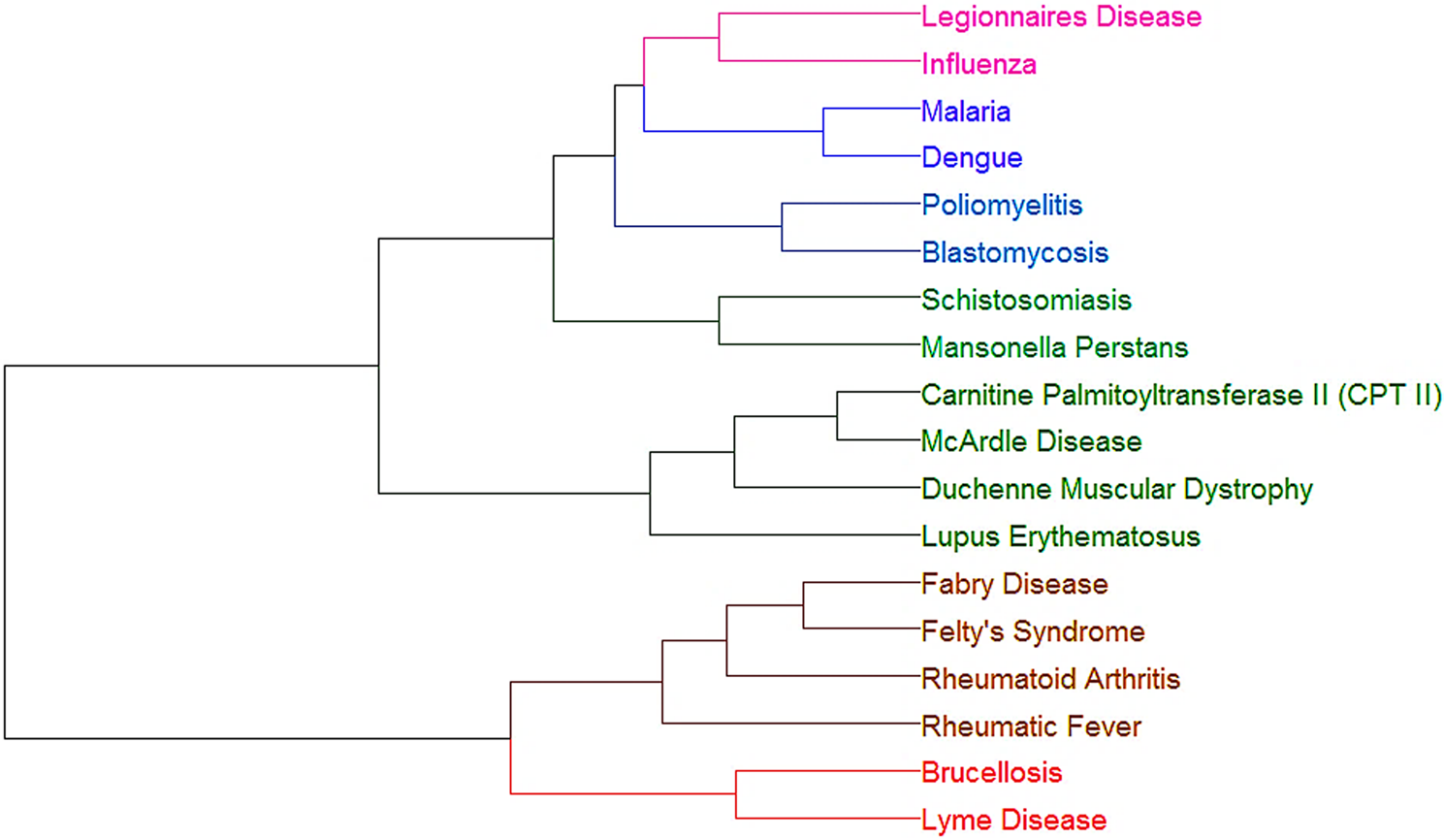
HCA using two symptoms as inputs: Myalgia and Joint Pain

In this initial analysis, we input two symptoms - myalgia and joint pain - and performed hierarchical cluster analysis (HCA), with results visualized in the dendrogram of Fig. 1. The dendrogram shows closer (more similar) diseases connected by lower positioned horizontal lines. It reflects patterns among diseases sharing these input symptoms. The HCA algorithm further joined in clusters diseases sharing keywords, represented with specific colours, based on symptom profiles.

Our database contained 133 diseases spanning common, less common, and rare diagnoses across various pathological groups. We have made the deliberate choice to assign equal weights to all symptoms and risk factors, intending to create an unbiased, unsupervised framework. This approach allows students to develop diagnostic reasoning based on their existing medical knowledge rather than preassigned importance of certain symptoms. Key observations include: infectious diseases (Legionnaires’ disease, influenza, malaria, dengue, Lyme disease, brucellosis) forming distinct but related clusters; autoimmune/genetic conditions (McArdle’s disease, CPT-II deficiency, Fabry disease, Felty syndrome) grouping by pathophysiology. While some clinically related infections (e.g., Lyme and Legionnaires’ diseases) appeared in separate clusters, the analysis successfully captured broader etiological relationships. The analysis also highlights diagnostically relevant patterns among rare conditions, particularly genetic disorders, and geographically restricted diseases (e.g., malaria, dengue, Lyme disease). This feature prompts students to consider epidemiological factors, including endemicity and environmental exposures, when formulating differential diagnoses, thereby reinforcing the importance of contextual clinical reasoning.

### Example 2- inputs: symptom1: abdominal pain; symptom2: diarrhoea and risk factor: smoking

**Fig. 2:**
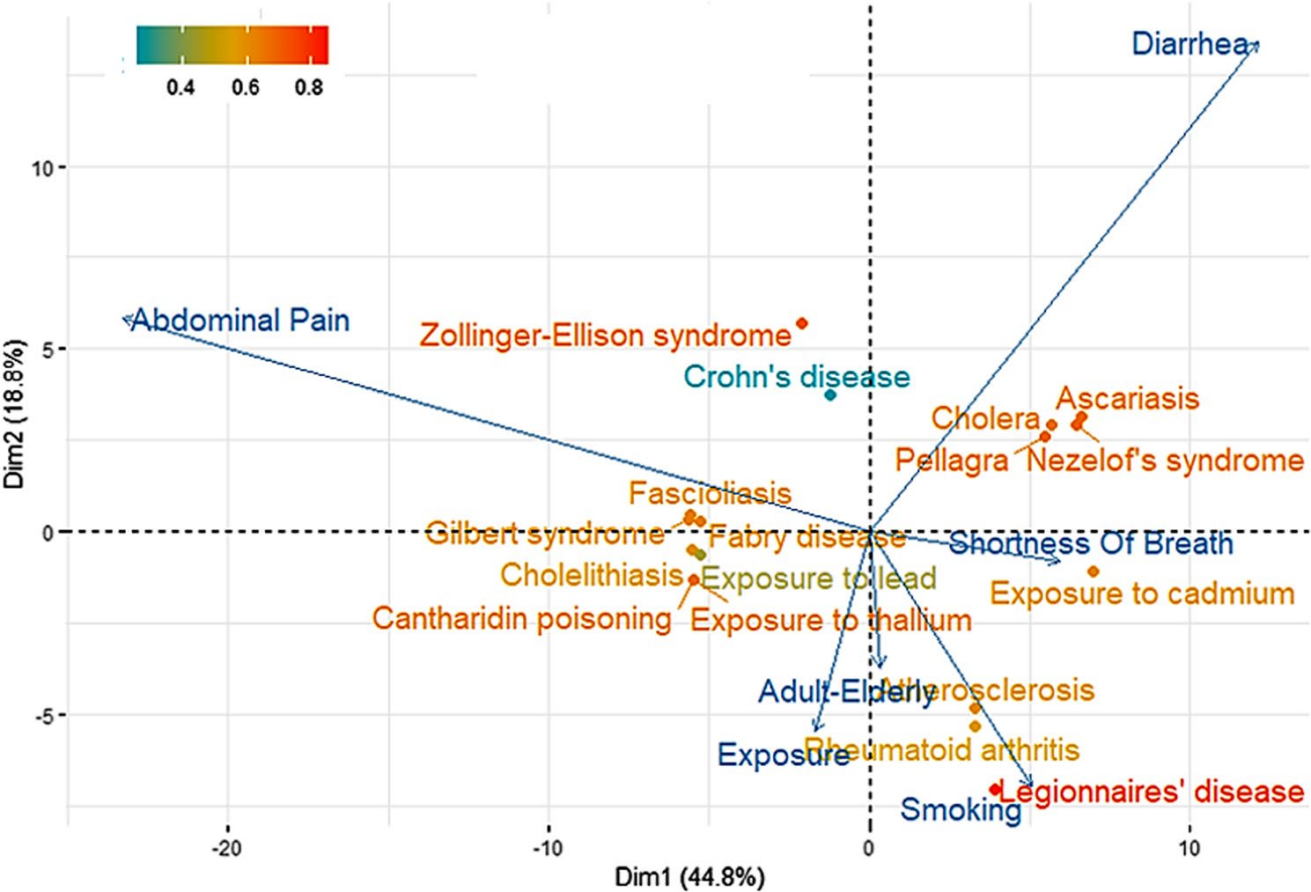
PCA using the inputs: Abdominal Pain, Diarrhoea and Smoking

The analysis employed abdominal pain, diarrhoea, and smoking as input variables for principal component analysis (PCA), with results shown in Fig. 2. Information retrieved corresponds to 44.8% in the first component and 18.8% in the second, which is deemed sufficient for providing adequate evidence. The biplot (diseases and symptoms/risk factors) illustrates relationships across the two primary dimensions, where arrows represent clinical variables (longer arrows indicate higher loadings i.e., a higher participation of the original variables in the transformed ones) and coloured dots indicate diseases, i.e. scores, graded by the quality of representation, from red [high] to blue [low]. The input symptoms showed expected associations: diarrhoea correlated strongly with cholera and pellagra, while abdominal pain linked to metal poisoning and Gilbert’s syndrome. Notably, non-input variables like environmental exposure and age emerged as potential diagnostic factors, whereas smoking (shorter arrow) demonstrated negligible correlation with the symptoms profile.

### Example 3 - inputs: symptom1: dyskinesia; symptom2: impaired gait and risk factor: depression

**Fig. 3:**
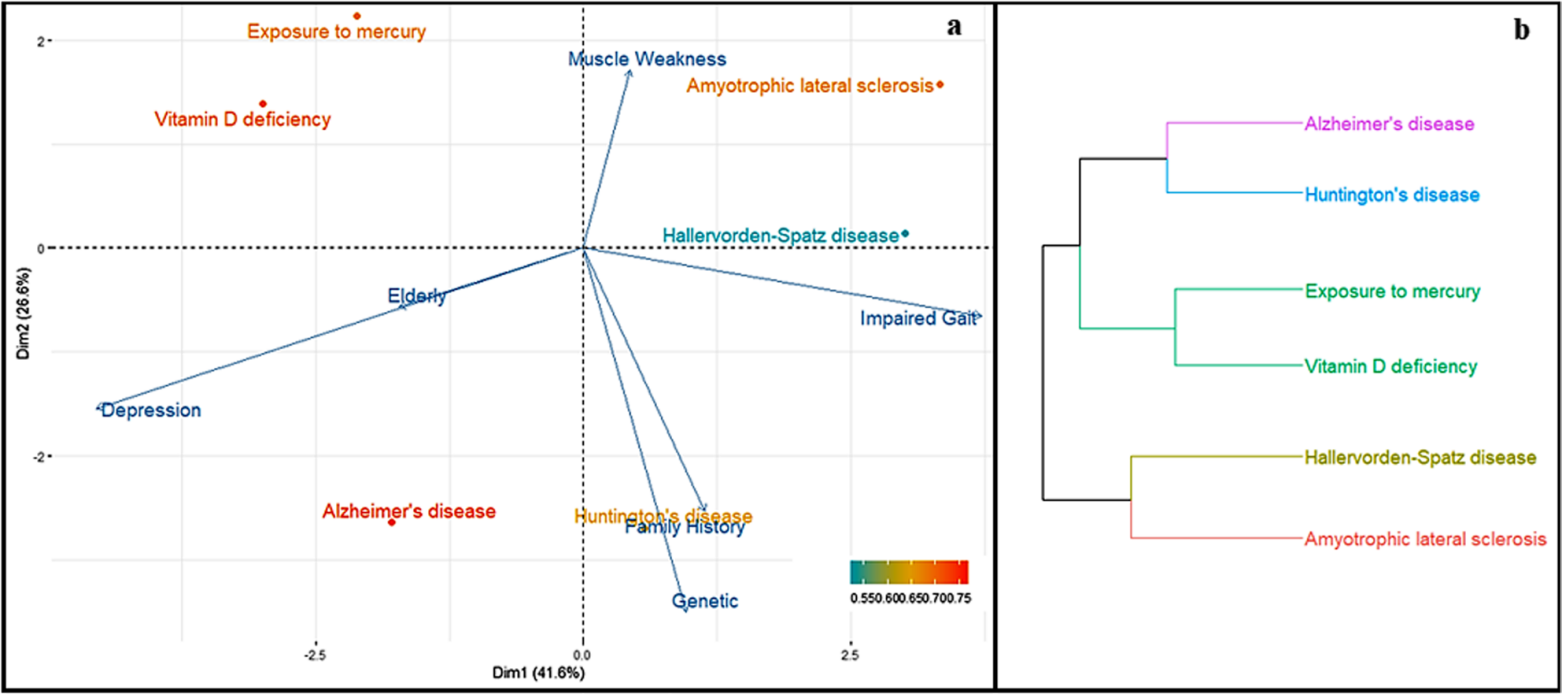
PCA (a) and HCA (b), using the inputs: Dyskinesia, Impaired Gait and Depression

The PCA results in Fig. 3a demonstrate distinct clinical associations, with muscle weakness showing strong loading for amyotrophic lateral sclerosis and family history emerging as a significant risk factor for Huntington’s disease. Concurrent hierarchical cluster analysis (HCA) revealed unexpected groupings, including a cluster containing mercury exposure and vitamin D deficiency, while other diseases showed minimal phenotypic overlap (Fig. 3b). This multivariate approach effectively discriminates between disease relationships, prompting medical students to conduct more in-depth diagnostic analysis.

We aim to encourage students to interpret PCA and HCA outcomes critically and reach their own conclusions. The goal is not to produce fully accurate clinical groupings —which would be necessary if the methodology were intended as a diagnostic tool — but rather to foster the development of students’ reasoning and critical thinking skills under the guidance of a teacher. By engaging with the data in this way, students learn to evaluate and question statistical outputs, improving their analytical and diagnostic reasoning.

## Discussion

The proposed exercise combines two complementary educational components in an exploratory design proposed as a new tool to teach and learn Medicine. First, database construction requires students to: (1) research diseases of interest, including less common conditions, for example consulting rare diseases data sources; [14] (2) identify key clinical descriptors through literature review; and (3) evaluate risk factors that may lack clear classification in standard guidelines, after they have captured the patient’s signs and symptoms. This process reinforces comprehensive disease understanding while developing information synthesis skills essential for clinical practice.

Second, script development cultivates computational thinking, regardless of programming language choice (R was used here for its statistical packages). While AI-assisted coding tools lower technical barriers, the core educational value lies in structuring logical workflows to analyse clinical patterns. Students subsequently validate their models by comparing computational outputs with established medical knowledge, creating a feedback loop that strengthens both diagnostic reasoning and data literacy.

The implemented examples demonstrate the methodology’s pedagogical utility by generating computational outputs that identify clinically relevant symptoms and risk factors, which students must critically evaluate for diagnostic reliability. This process simultaneously trains clinical reasoning skills, emphasizing the importance of targeted history-taking - particularly for environmental exposures, travel history, or toxic substance contact in endemic regions. The proposed method’s adaptable design accommodates varying student competencies and supports both independent and collaborative learning, making it suitable for integration across medical education curricula. Further studies are needed to better understand the value of the approach proposed in this paper and its implications for medical education.

## Conclusion

This methodology provides a dual-focused educational exercise combining: (1) literature-based development of clinical knowledge through database construction, and (2) critical evaluation of computational outputs to refine diagnostic reasoning. The approach not only enhances awareness of less common diseases and environmental risk factors but also cultivates essential digital competencies, including basic programming and AI-assisted coding skills - increasingly relevant for modern medical practice. By integrating these transversal scientific skills with core medical training, the method aligns with the evolving needs of digitally-native medical students and their future practice environments.

Medical schools worldwide employ diverse teaching methodologies, and the determination of which level of medical students would most benefit from this exercise is best made locally by each institution, based on their curricular structure and pedagogical approach. The manuscript provides a flexible framework that can be adapted to different levels and contexts, allowing educators to integrate the methodology according to their own educational objectives.

## Supplementary Information

Supplementary Material 1.

Supplementary Material 2.

## Data Availability

The database and computational script are available in the supplementary material.

## Abbreviations

PCA: Principal Component Analysis
HCA: Hierarchical Cluster Analysis
AI: Artificial Intelligence
